# Home Range of the Endangered Beale's Eyed Turtle ( *Sacalia bealei* ) and the Implications for Conservation

**DOI:** 10.1002/ece3.71520

**Published:** 2025-06-11

**Authors:** Xiangyu Yuan, Qingru Hu, Rongping Bu, Jiangbo Yang, Liu Lin, Hai‐Tao Shi

**Affiliations:** ^1^ Ministry of Education Key Laboratory for Ecology of Tropical Islands, Key Laboratory of Tropical Animal and Plant Ecology of Hainan Province College of Life Sciences, Hainan Normal University Haikou China; ^2^ Liuzhou No. 2 High School Liuzhou China; ^3^ College of Marine Science, Guangxi Key Laboratory of Beibu Gulf Biodiversity Conservation, Beibu Gulf University Qinzhou China; ^4^ Guangzhou CAOMUFAN Ecological Research co., Ltd. Guangzhou China

**Keywords:** Bayesian analysis, Beale's eyed turtle, Cliff's delta, home range, radiotelemetry, turtle conservation

## Abstract

The Beale's Eyed Turtle ( *Sacalia bealei* ) is endemic to China and endangered primarily due to poaching and habitat loss. However, limited ecological information for this species hinders conservation actions. Using radiotelemetry, we determined the home range size of *S. bealei* in its natural habitat, collecting 769 valid activity locations from nine turtles (3 males, 6 females). The line home range size (LHR), home range area (HR, 95% minimum convex polygons), and core home range area (CHR, 50% fixed kernel density estimation) were about 185 m, 0.626 ha, and 0.089 ha, respectively. Bayesian and Cliff's delta analyses revealed no significant sex‐based differences in LHR, HR, or CHR overall or during the non‐breeding season. However, during the breeding season, females exhibited significantly larger HR and CHR than males (Cliff's delta = 1, 95% CI [0.14, 1]; Bayes factors 2.06–3.27), reflecting increased movement for breeding. Home range overlap ranged from 0.30 to 0.33 across pair types annually, with no significant differences between the breeding and non‐breeding periods (Mann–Whitney U tests, *p* > 0.05). By highlighting species' vulnerability to habitat fragmentation and anthropogenic pressure, our findings emphasize the urgent need for strategic conservation interventions.

## Graphical abstract

This study examines the home range and spatial ecology of the endangered Beale's Eyed Turtle ( *Sacalia bealei* ) in China using radiotelemetry. We found that females had significantly larger home ranges during the breeding season compared to males, while no sex‐based differences were observed in the non‐breeding season. Our results underscore the species' vulnerability to habitat fragmentation and highlight the need for targeted conservation strategies to mitigate anthropogenic pressures.

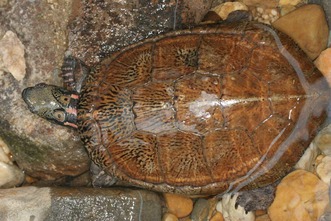

## 1 Introduction

More than 60% of turtle species are currently endangered, making them one of the most endangered groups of animals globally (Rhodin et al. 2018). The high percentage of threatened turtle species highlights the critical need for conservation initiatives to prevent their extinction (Lovich et al. 2018; Stanford et al. 2020). However, ecological research on turtle species is limited. Asia is a turtle diversity hotspot yet faces significant gaps in ecological data largely because of the scarcity of stable wild populations (Buhlmann et al. 2009; Shen et al. 2010). Addressing these gaps is critical for effective conservation strategies because a thorough understanding of wild turtle ecology can inform habitat protection and population recovery efforts.

Home range, a key measure in spatial ecology, represents the total area covered by an individual while performing essential activities, such as foraging, mating, and nesting (Burt 1943). This metric influences the ability of an organism to secure resources, affects its survival, and may indicate its physical condition. Additionally, the relationship between home range size and body size has been used to assess resource availability and habitat quality and to inform land management practices and the size of protected areas (Madden 2023).

**FIGURE 1:**
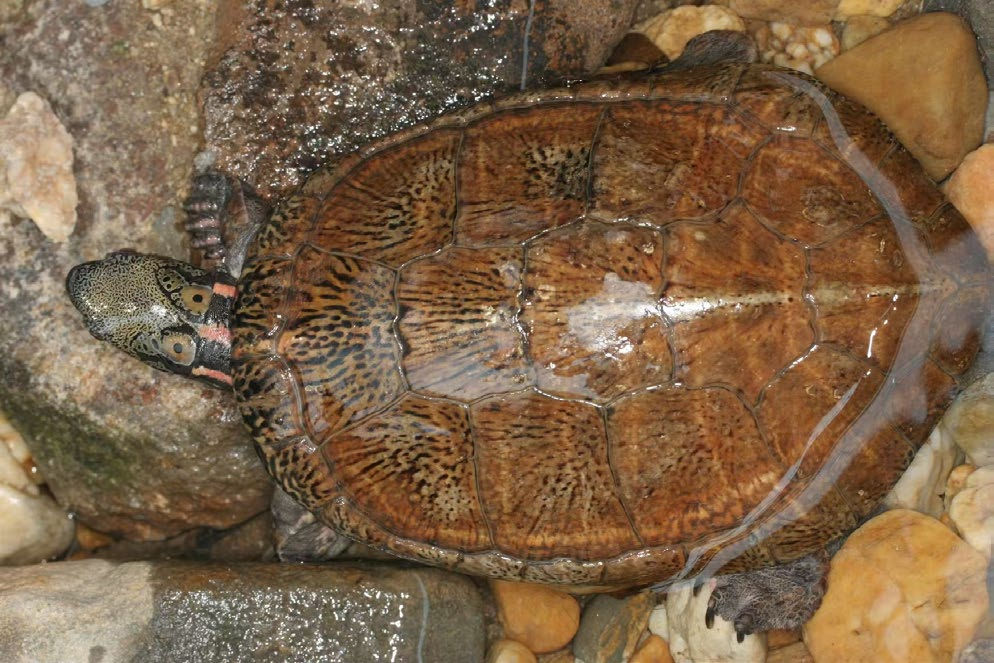
A male Beale's Eye Turtle ( *Sacalia bealei* ). Photo credit: Hai‐Tao Shi.

The Beale's Eyed Turtle ( *Sacalia bealei* ; Figure 1) is found in southern China, including provinces such as Guangdong, Fujian, Anhui, Hunan, Jiangxi, Guizhou, and Hong Kong (Shi et al. 2008; Lin et al. 2022). Classified as endangered on the IUCN Red List (IUCN 2000), its CITES protection status increased from Appendix III to Appendix II in 2013. Since 2021, this species has been granted second‐class national protection, although this protection only applies to wild populations, leaving captive‐bred individuals less regulated. Major threats to *S. bealei* include poaching and habitat loss (Lin et al. 2018), which are exacerbated by the food industry, traditional medicine, and the pet trade (Hu et al. 2016). The declining population has made it increasingly difficult to locate wild study groups with sufficient sample sizes, limiting the collection of crucial ecological information such as habitat preferences and home range sizes, and hampering research on the environmental needs of this species in the wild.

The current study aimed to quantify the home range of wild *S. bealei* in its natural, undisturbed habitat in southern China. Combined with our previous findings on habitat selection (Hu et al. 2016) and reproductive ecology (Lin et al. 2018), we aim to provide a complete picture of how these turtles utilize their environments. This will enable more effective habitat management and protection strategies to support the long‐term conservation of this endangered species.

## 2 Materials and Methods

### 2.1 Study Site

**FIGURE 2:**
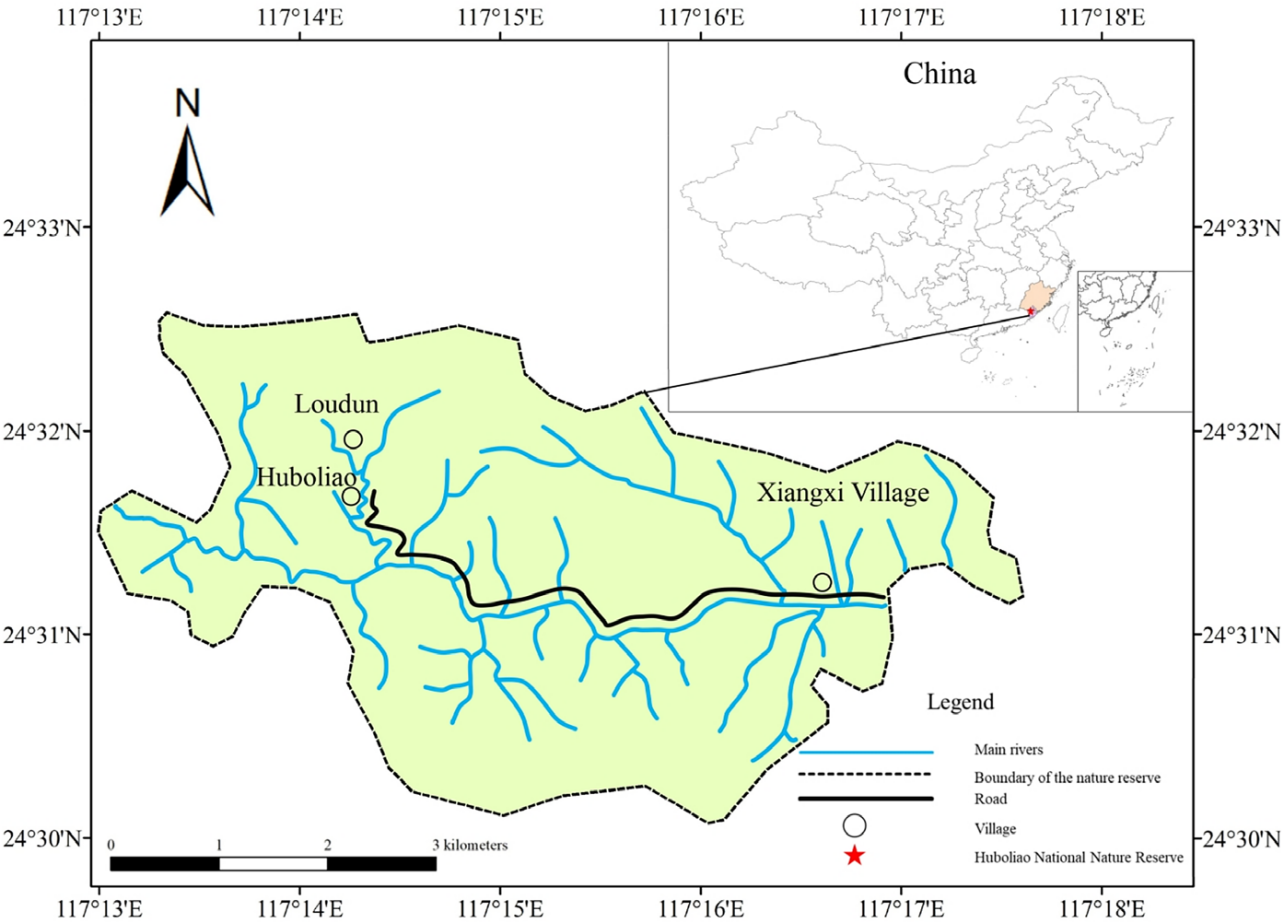
Map of the study area.

The study was conducted in the Huboliao National Nature Reserve in Nanjing County, Fujian Province, China (Figure 2). This area features low‐to mid‐altitude mountains ranging from 137 to 875 m and numerous dendritic streams. Due to limited local monitoring, climate data from Nanjing County (2014–2015) were used, with annual temperatures of 21.3°C–21.5°C, annual rainfall of 1643–2054 mm, and annual relative humidity of 80% (Zhangzhou Municipal Bureau of Statistics 2014, 2015). The vegetative community includes southern subtropical evergreen broad‐leaved forests, mixed coniferous‐broadleaf, and bamboo forests (Fan 2001).

### 2.2 Turtle Capture and Morphometric Measurements

We studied the home ranges of ten wild adult *S. bealei* , comprising three males and seven females, using radio telemetry from November 2014 to October 2015. One female (ID 2) was excluded from the final analysis due to incomplete tracking data caused by transmitter malfunction. These turtles were captured using cylindrical nylon traps (55 cm length ×25 cm diameter) baited with salted fish and meat in natural streams (Hu et al. 2016). All individuals were brought to the study base for measuring their morphometric characters using vernier calipers (accuracy: ± 0.1 mm, Mitutoyo Corporation, Kawasaki, Japan) and weighing their mass using an electronic balance (accuracy: ± 1 g, Guangdong Senssun Electronics Co. Ltd., Zhongshan, China). Morphometric measurements were taken as follows: Carapace Length (CL): the maximum straight‐line distance between the front of the cervical and the rear of the supracaudal scutes; Carapace Width (CW): the maximum straight‐line distance between the two sides of the carapace; Carapace Height (CH): the maximum height of the shell; Plastron Length (PL): the maximum straight‐line distance between the front and rear edges of the plastron; and Plastron Width (PW): the maximum straight‐line distance between the two sides of the plastron (Shi et al. 2013). Adult *S. bealei* were identified based on sexually dimorphic morphological characteristics described by Lin et al. (2017). Females were distinguished by a carapace length exceeding 120 mm, corresponding to the size of the smallest known gravid female (117–120 mm) and by specific coloration patterns, including yellow posterior ocelli on the head, faint yellow neck stripes, and fewer but larger black patches on the plastron. Males were identified by the presence of bright‐red neck stripes, supplemented by additional traits such as brown posterior ocelli on the head, numerous tiny black dots on the head, and small black spots on the peripheral area of the plastron.

### 2.3 Radio Telemetry

Each turtle was equipped with a 6‐g radio tracking transmitter (RI‐2B,216.000–216.999 MHz; Holohil Systems Ltd., Ottawa, ON, Canada; battery life of 12–24 months) attached to the posterior carapace using epoxy resin. The total weight of the transmitter and adhesive was less than 8% of the turtle's body weight (Pike 2006). After the transmitters were attached, the turtles were returned to their original capture sites. Tracking was initiated one week after capture to minimize handling effects on the turtles' behavior, ensuring data collection under normalized conditions. Tracking was conducted twice daily, in the morning (06:00–10:00) and afternoon (14:00–18:00). Turtle locations were recorded to 3–5 m accuracy using a handheld GPS device (Magellan Triton 400E) under optimal conditions of signal reception. Upon completion of the study, all turtles were recaptured, their transmitters were removed, and they were released at their original capture locations.

### 2.4 Definition of Breeding and Non‐Breeding Periods

Breeding and non‐breeding periods for *S. bealei* home range analyses were defined based on its reproductive ecology, informed by telemetry, field observations of nesting, and breeder interviews (Lin et al. 2018). The breeding period (April–June, November) included mating (April, November) and egg‐laying (late April–mid‐June), reflecting active reproductive behaviors (Carr 1995). November mating supports sperm storage for spring egg‐laying, common in turtles (Kuchling 1998). The non‐breeding period (July–October) included incubation and hatching, which are environmentally driven developmental phases without parental involvement, unlike active reproduction, plus foraging and basking. Hibernation occurred from December to March, when turtles were inactive. This delineation aligns with chelonian reproductive biology, where reproductive effort ends after oviposition (Carr 1995; Kuchling 1998).

### 2.5 Home Range and Overlap Index Calculation

Home range areas were calculated using Home Range Tools in ArcGIS 9.3. Specifically, home range (HR) areas were determined using 95% minimum convex polygons (95% MCPs; Anderson 1982; Bekoff and Mech 1984), and core home range (CHR) areas were calculated using a 50% fixed kernel density estimation (50% FKDE; Donaldson and Echternacht 2005). Line home range (LHR) size was defined as the straight‐line distance between the two most distant GPS points (Sexton 1959; Pluto and Bellis 1988). To assess variation in spatial interactions across reproductive periods, home range overlap indices were compared between the breeding and non‐breeding periods using non‐parametric statistical tests. The overlap index (OI) was used to quantify the degree of overlap between the home ranges of two individuals based on the size of their 95% MCP home ranges. The overlap index ranges from 0 to 1, with larger values indicating a higher degree of overlap (Atwood and Weeks Jr. 2003).

### 2.6 Statistical Analysis

Data analyses were conducted in R (v4.4.3) using the BayesFactor and effsize packages, with supplementary analyses performed in SPSS (v19.0). Given the small sample sizes (*n* = 3–6) for LHR, HR, and CHR comparisons, and the potential violation of normality, traditional parametric (e.g., t‐tests) and non‐parametric (e.g., Mann–Whitney U) tests were considered unsuitable. Parametric tests assume normality, while Mann–Whitney U tests may lack power with very small samples (*n* < 5) and skewed data (Helsel and Hirsch 2002). Therefore, the following analytical approaches were adopted:

1. A Bayesian independent samples t‐test via the ‘ttestBF’ function (BayesFactor package) with a default Jeffreys‐Zellner‐Siow (JZS) prior (*r* = 0.707), supplemented by sensitivity analyses using conservative (*r* = 0.5) and liberal (*r* = 1.0) priors (Rouder et al. 2009).
2. Cliff's delta (*δ*) was used to estimate non‐parametric effect sizes. It was also applied to comparisons of home range overlap indices when appropriate (between periods).
3. Mann–Whitney U tests were used for comparisons involving larger sample sizes (*n* = 15–36), particularly for home range overlap comparisons between breeding and non‐breeding periods. Statistical significance was set at *α* = 0.05.

#### 2.6.1 Interpretation of Bayesian *t*‐Tests

The Bayesian independent samples t‐test was selected for its robustness with small sample sizes and non‐normally distributed data. Unlike traditional methods, this test does not rely on normality assumptions and provides a direct measure of evidence for the alternative hypothesis through the Bayes Factor (BF_10_). A BF_10_ greater than 3 was interpreted as indicating moderate evidence, while values between 1 and 3 indicated weak evidence for group or period‐based differences (Jeffreys 1961; Andraszewicz et al. 2014).

#### 2.6.2 Interpretation of Cliff's Delta

Cliff's delta (*δ*; Cliff 1993) quantifies the extent of non‐parametric distributional dominance between two groups on a scale from −1 to +1, where 0 indicates no difference. It was calculated using the cliff.delta function in R, with |*δ*| values ≥ 0.15, 0.33, and 0.47 interpreted as small, medium, and large effects, respectively (Meissel and Yao 2024). Effects with 95% confidence intervals that included zero were considered statistically non‐significant (Hogarty and Kromrey 1999).

## 3 Results

The morphometric characteristics of captured *S. bealei* specimens are summarized in Table 1. Across different prior scales, BF_10_ showed consistent patterns (Table S1), indicating robustness in statistical inference.

**TABLE 1:** Morphometric data of *Sacalia bealei* captured during this study.

ID	Sex	BW (g)	CL (mm)	CW (mm)	PL (mm)	PW (mm)	CH (mm)
1	♀	362	141.5	100.1	124.7	84.8	57.6
3	♂	339	144.5	96.4	129.6	79.6	48.3
4	♀	391	144.5	96.5	122.1	80.0	52.3
5	♀	384	150.2	106.9	135.8	87.1	53.5
6	♂	275	128.9	91.9	111.6	78.7	47.6
7	♀	442	150.9	104.9	134.4	86.0	54.9
8	♀	245	132.5	93.0	115.1	77.6	42.8
9	♂	397	138.6	99.7	122.2	82.9	51.6
10	♀	385	148.3	97.0	133.4	78.6	51.6

Abbreviations: BW, body weight; CH, Carapace Height; CL, Carapace length; CW, Carapace width; PL, Plastron length; PW, Plastron width.

### 3.1 Line Home Range and Home Range Area

**FIGURE 3:**
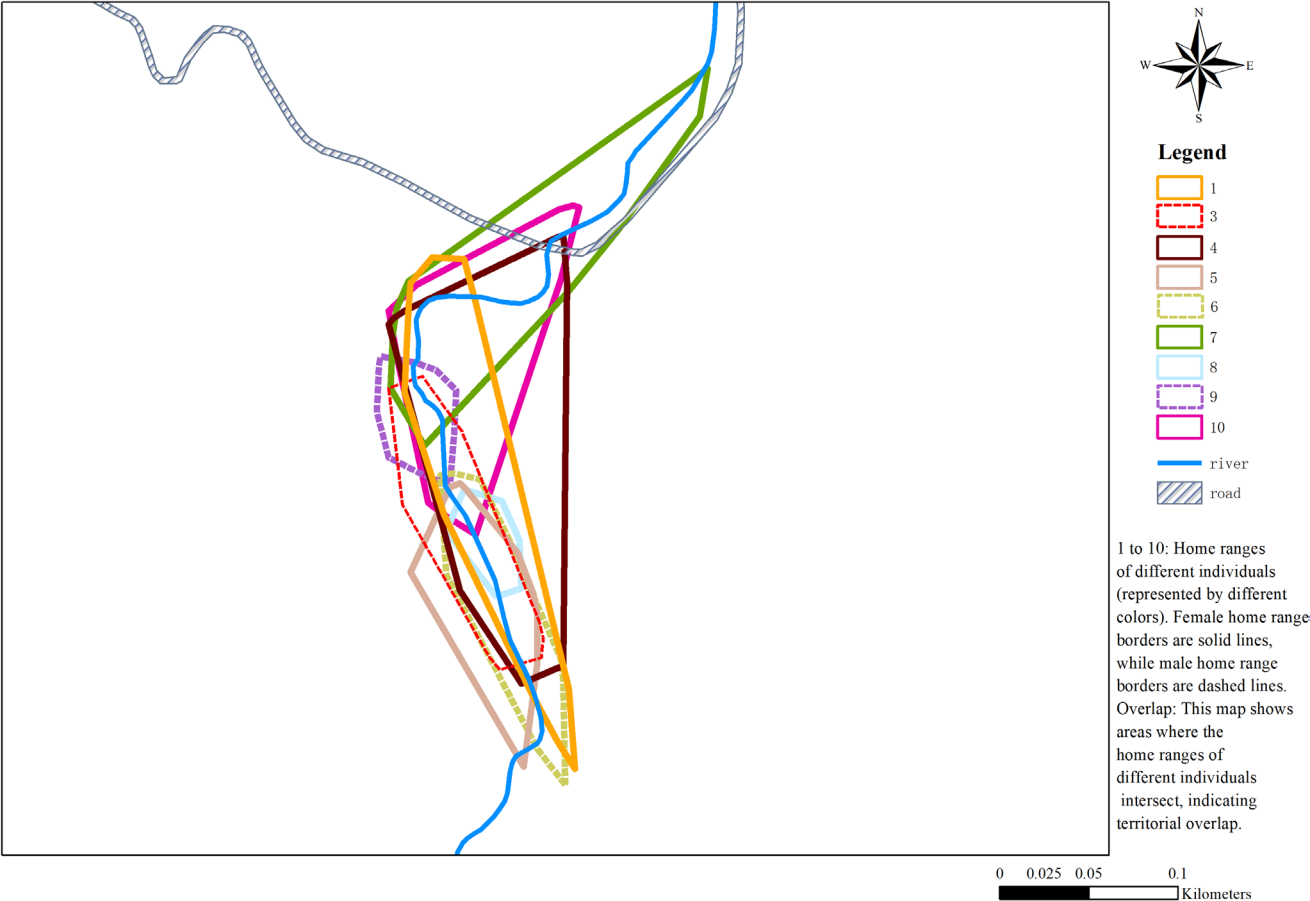
Home range of nine *Sacalia bealei* individuals over a full year. Polygons of different colors represent the home range of different individuals. Female home range borders are solid lines, while male home range borders are dashed. This map also shows areas where the home ranges of different individuals intersect, indicating territorial overlap.

A total of 769 valid locations of nine *S. bealei* were recorded via telemetry from November 2014 to October 2015. The LHR of *S. bealei* was 185 ± 26 (range 62–295) m, HR area was 0.626 ± 0.14 (range 0.081–1.411) ha, and CHR area was 0.089 ± 0.02 (range 0.019–0.208) ha (Table 2, Figure 3).

**TABLE 2:** Line and area home range of *Sacalia bealei* during the study period (November 2014–October 2015).

ID	Sex	Locations	LHR (m)	HR (ha)	CHR (ha)
1	♀	81	295	0.915	0.208
4	♀	82	251	1.411	0.158
5	♀	112	162	0.326	0.046
7	♀	88	255	0.899	0.105
8	♀	65	62	0.081	0.019
10	♀	75	190	0.873	0.107
Mean ± SE			203 ± 34	0.751 ± 0.194	0.107 ± 0.03
3	♂	103	187	0.528	0.068
6	♂	93	184	0.401	0.057
9	♂	70	78	0.198	0.036
Mean ± SE			150 ± 36	0.375 ± 0.1	0.054 ± 0.009
Mean ± SE			185 ± 26	0.626 ± 0.14	0.089 ± 0.02
BF_10_	♀ vs. ♂		0.68	0.82	0.81
Cliff's delta	♀ vs. ♂		0.44	0.44	0.44

Bayesian independent samples t‐tests revealed no substantial evidence for sex‐based differences in LHR, HR, or CHR, with BF_10_ consistently below 1. While Cliff's delta indicated moderate‐to‐large effect sizes (*δ* = 0.44), the 95% confidence intervals (CIs) included zero, suggesting that these effects were not statistically significant at the *α* = 0.05 level (Table S1).

### 3.2 Difference in Home Range Between Breeding and Non‐Breeding Seasons of *S. bealei*

We analyzed data from three females and three males during breeding and non‐breeding seasons to ensure balanced sample sizes with complete telemetry records. Three additional females were excluded due to incomplete data across reproductive periods (e.g., transmitter malfunction or signal loss), which could bias temporal comparisons. This approach minimizes confounding effects from missing values. The differences in HR size between female (3♀) and male (3♂) *S. bealei* during the breeding and non‐breeding seasons were measured. During the non‐breeding season, Bayesian independent samples t‐tests for LHR, HR, and CHR provided weak evidence for differences between female and male groups, with BF_10_ ranging from approximately 0.57 to 1.07. Cliff's delta values ranged from small to moderate‐to‐large (*δ* = −0.11 to 0.44); however, the 95% confidence intervals for all three variables included zero, indicating no statistically significant differences at the 0.05 level. During the breeding season, the Bayesian independent samples t‐test for LHR provided weak evidence for a difference between female and male groups (BF_10_ ≈ 1.1). Cliff's delta indicated a large non‐parametric effect (*δ* = 0.78); however, the corresponding 95% confidence interval included zero, indicating a lack of statistical significance at the 0.05 level. For HR and CHR during the breeding season, BF_10_ ranging from approximately 2.06 to 3.27 provided weak to moderate evidence for sex‐based differences. Cliff's delta indicated the largest possible non‐parametric effect (*δ* = 1), and the corresponding 95% confidence interval did not include zero ([0.14, 1]), suggesting that these effects were statistically significant at the 0.05 level (Table 3).

**TABLE 3:** Difference in home range between female and male *Sacalia bealei* in breeding and non‐breeding seasons.

Season	ID	Sex	LHR (m)	HR (ha)	CHR (ha)
Non‐breeding season	4	♀	67	0.059	0.015
	7	♀	71	0.129	0.031
	10	♀	72	0.129	0.018
	Mean ± SE		70 ± 1	0.106 ± 0.02	0.021 ± 0.005
	3	♂	44	0.072	0.016
	6	♂	72	0.143	0.029
	9	♂	51	0.119	0.025
	Mean ± SE		56 ± 8	0.111 ± 0.02	0.023 ± 0.004
	BF_10_	♀ vs. ♂	1.05	0.57	0.58
	Cliff's delta	♀ vs. ♂	0.44	−0.11	−0.11
Breeding season	4	♀	132	0.420	0.075
	7	♀	232	0.835	0.135
	10	♀	149	0.713	0.128
	Mean ± SE		171 ± 31	0.656 ± 0.12	0.113 ± 0.02
	3	♂	104	0.250	0.053
	6	♂	143	0.248	0.061
	9	♂	50	0.112	0.027
	Mean ± SE		99 ± 27	0.203 ± 0.05	0.047 ± 0.01
	BF_10_	♀ vs. ♂	1.10	2.87	2.31
	Cliff's delta	♀ vs. ♂	0.78	1	1

### 3.3 Home Range Overlap

Home range overlap indices (Volume of Intersection) were calculated for all individual pairs within the study population throughout the year. The overall average pairwise home range overlap index for the entire year was 0.32 ± 0.24 (*n* = 36). When categorized by pair type for the entire year, average overlap indices were: Male–Male pairs: 0.33 ± 0.34 (*n* = 3), Male–Female pairs: 0.30 ± 0.24 (*n* = 18), and Female–Female pairs: 0.33 ± 0.24 (*n* = 15). During the breeding period, the overall average home range overlap index across all pairs was 0.14 ± 0.16 (*n* = 36), with pair‐type averages as follows: Male–Male pairs: 0.03 ± 0.05 (*n* = 3), Male–Female pairs: 0.14 ± 0.14 (*n* = 18), and Female–Female pairs: 0.16 ± 0.19 (*n* = 15). In the non‐breeding period, the overall average home range overlap index decreased to 0.11 ± 0.20 (*n* = 36). The indices were 0 for Male–Male pairs (*n* = 3), 0.19 ± 0.26 (*n* = 18) for Male–Female pairs, and 0.03 ± 0.07 (*n* = 15) for Female–Female pairs. Non‐parametric comparisons using Mann–Whitney U tests revealed no statistically significant differences in home range overlap indices between the breeding and non‐breeding periods for Overall, Male–Female, and Female–Female dyads (Table 4). For Male–Male dyads, both Bayesian analysis and Cliff's delta indicated no statistically significant difference (BF_10_ = 0.65–0.80; *δ* = 0.33; 95% CI = [−0.51, 0.85]; Table S1).

**TABLE 4:** Home range overlap indices across different periods and pair types.

Period	Overall mean ± SD (*n* = 36)	Male–male mean ± SD (*n* = 3)	Male–female mean ± SD (*n* = 18)	Female–female mean ± SD (*n* = 15)
Year‐round	0.32 ± 0.24	0.33 ± 0.34	0.30 ± 0.24	0.33 ± 0.24
Breeding	0.14 ± 0.16	0.03 ± 0.05	0.14 ± 0.14	0.16 ± 0.19
Non‐breeding	0.11 ± 0.20	0	0.19 ± 0.26	0.03 ± 0.07
P (Breeding vs. Non‐breeding)	0.165	See Table S1	0.868	0.128

## 4 Discussion

The HR of *S. bealei* was measured at 0.626 ha, significantly exceeding the 0.002 ha range reported by Cheung (2007) for a single individual in Tai Po Kau Forest, Hong Kong, based on a 20‐month telemetry study. This difference may be attributed to the smaller and more fragmented habitat in Hong Kong, where resources are likely concentrated in a limited area, reducing the need for the individual to expand its HR. Additionally, the low population density and environmental pressures in Hong Kong may further restrict individual movement. Individual variations in behavior, such as a preference for limited movement or site fidelity, could also contribute to the observed difference in HR size. *Sacalia bealei* inhabits mountain streams, and its habitat is comparable to that of the Big‐headed Turtle ( *Platysternon megacephalum* ), which has an HR of nearly 0.1 ha (Sung et al. 2015). However, these HRs are substantially smaller than those of other freshwater turtles in rivers, lakes, and swamps. For instance, Reeves' Turtles ( *Mauremys reevesii* ) in Qichun County, Hubei Province, China, have an HR of 14.34 ha (Bu et al. 2023). Similarly, the Central Chiapas Mud Turtle ( *Kinosternon abaxillare* ) in Mexico has HRs of 2.34 ha for females and 5.18 ha for males (Reyes‐Grajales and Macip‐Rios 2023). Moreover, the Chinese Softshell Turtle ( *Pelodiscus sinensis* ) in the Yellow River, Northwestern China, has an HR of 1.36 ha (Kong et al. 2021). Using the 50% fixed kernel method, the CHR of *S. bealei* was estimated at 0.089 ± 0.02 ha, indicating concentration in specific stream segments. This concentration is likely associated with ecological needs such as mating and foraging. In resource‐abundant areas, turtles may not need to cover large areas to find food or mates (Brown et al. 1994; Litzgus and Mousseau 2004), resulting in smaller HRs.

The absence of significant sex‐based differences in annual HR size may be attributed to site fidelity and similar physiological needs outside the breeding season (Ernst 1970; Jones 1996; Carter et al. 1999; Morrow et al. 2001). During the breeding season, females exhibited significantly larger HR and CHR than males, as evidenced by a large non‐parametric effect (Cliff's delta = 1, 95% CI [0.14, 1] for HR; CI for CHR not containing zero). Bayesian analyses provided support for these differences (BF_10_: 2.06–3.27), reinforcing the statistical significance. This suggests that females expand their ranges for reproductive activities, consistent with patterns in other species. For example, Litzgus and Mousseau (2004) observed that gravid female Spotted Turtles ( *Clemmys guttata* ) had significantly larger HRs (19.06 ha) than males (5.15 ha). Gibbons et al. (1990) proposed that long‐distance movement is influenced by energy expenditure and predation risk. Under abundant resources and suitable habitat conditions, turtles may maintain small HRs, with females increasing their ranges only during nesting. In the present study, the sex‐based differences in HR and CHR observed during the breeding season were not statistically significant during the non‐breeding season, suggesting similar habitat use by males and females when reproductive pressures were absent.

Although sex‐based differences in HR and CHR were not statistically significant overall or during the non‐breeding season, females exhibited larger mean home range sizes. The pronounced sexual dimorphism in *S. bealei* , in which females are larger than males (Lin et al. 2017), may explain their larger HRs, as body size is a crucial determinant of HR size in turtles (Slavenko et al. 2016). McNab (1963) indicated that an animal's activity range is proportional to its weight, owing to its metabolic requirements. Larger animals require more energy and thus require larger foraging areas. Although larger animals may have overlapping activity ranges, energy demand significantly influences the extent of their ranges (Jetz et al. 2004). Under the conditions of abundant food resources, a well‐maintained habitat structure, and a suitable climate within the protected area, *S. bealei* tended to maintain relatively small HRs.

Home range overlap refers to the extent to which the spatial areas utilized by different individuals or groups coincide (Powell and Mitchell 2012). The overlap between the HRs of individuals can provide a valuable understanding of how they share space, which is essential for understanding patterns of social interactions, resource utilization, and disease transmission risk (Fieberg and Kochanny 2005). Notably, the average overlap indices were relatively consistent across all pair types for the entire year, ranging from 0.30 for Male–Female pairs to 0.33 for both Male–Male and Female–Female pairs. Despite these numerical differences between the breeding and non‐breeding seasons, statistical analyses (Mann–Whitney U tests, Bayesian, and Cliff's delta analyses) revealed no significant differences in overlap for Overall, Male–Female, Female–Female, and Male–Male pairs. These values suggest that individuals frequently utilize shared spatial areas throughout the year (Fieberg and Kochanny 2005); this also suggests that the observed degree of home range overlap is a general characteristic of spatial relationships within this population, rather than being specific to particular sex combinations. This shared space use, consistently observed across different pair types, implies a spatial organization where individuals routinely utilize overlapping areas, potentially creating opportunities for social interactions (Powell and Mitchell 2012). This spatial proximity may support essential social behaviors such as mating and communication, further highlighting the importance of conserving intact, interconnected habitats (Harless et al. 2009; Hughes et al. 2023). For *S. bealei* , such aggregation may enhance reproductive success by increasing mate encounter rates. However, overlapping ranges also indicate that habitat fragmentation can significantly disrupt social structure and reproductive success, as concentrated spatial use increases vulnerability to anthropogenic disturbances.

During the study, we also documented the presence of *Platysternon megacephalum* within the reserve. The occurrence of this species in the same habitat as *S. bealei* provides additional insights into the ecological composition of the area. *Platysternon megacephalum* , like *S. bealei* , is a stream‐dwelling turtle that relies on pristine aquatic environments for survival. Its presence further highlights the ecological significance of the reserve and underscores the importance of habitat conservation efforts to protect both species. Future studies should investigate these species' interactions and habitat requirements to inform more comprehensive conservation strategies.

Moreover, the high economic value of *S. bealei* in the pet trade and for traditional medicine has exacerbated the pressure of illegal hunting (Shi et al. 2008; Lin et al. 2018). The low‐altitude distribution of *S. bealei* overlaps with regions of intense human activity, making it more accessible to poachers. With such restricted and exploited HRs, localized populations can be easily decimated by targeted poaching, leading to rapid decline and even local extirpation. The combination of restricted movement, habitat loss, and exploitation highlights the urgent need for comprehensive conservation strategies.

Effective conservation measures should include protecting and restoring riparian forests to enhance habitat quality and connectivity (U.S. Fish and Wildlife Service 2024). Establishing buffer zones along streams may reduce the impact of human activity and provide critical foraging and nesting areas (Semlitsch and Bodie 2003). Strengthening the enforcement of anti‐poaching laws and raising public awareness about the endangered status of this species are also crucial steps. Community‐based conservation programs have successfully involved local populations in protection efforts, thereby reducing illegal hunting (Cheloti and Mulu 2023), and these efforts should be expanded.

The present study on the HR of *S. bealei* provides essential ecological information, yet there were some limitations. First, the relatively small sample size, involving only nine individual turtles, could limit the applicability of the results to larger populations or other habitats. Second, the study focused on a single geographic location (the Huboliao National Nature Reserve), which may not fully represent the habitat variability across the species' entire range. In addition, while behavioral and habitat‐use differences between the breeding and non‐breeding periods were analyzed, further longitudinal studies with larger sample sizes are needed to capture potential inter‐annual variation. While effective, the use of radio telemetry may also introduce some bias due to the potential influence of the transmitter on the turtles' behavior. Despite these limitations, the findings have significant implications for conservation strategies. Specifically, the small HRs and overlapping territories highlight the species' vulnerability to habitat fragmentation and anthropogenic pressures. Conservation measures should prioritize the maintenance of intact, continuous habitats and reduce human disturbances to enhance the viability of *S. bealei* populations. The combined application of Bayesian inference, Cliff's delta effect size estimation, and non‐parametric hypothesis testing enabled robust interpretation of spatial behavior despite small sample sizes and demonstrated a reproducible framework for ecological studies on rare or data‐limited species.

Additionally, community‐based conservation programs and stronger anti‐poaching measures are critical for ensuring the long‐term survival of this endangered species. Furthermore, given the small size of ranges, creating a network of protected areas covering the critical habitats of several local populations could be an effective strategy. Joint research efforts are required to monitor population trends and assess protective measures.

## Author Contributions

**Xiangyu Yuan:** data curation (equal), writing – original draft (lead), writing – review and editing (lead). **Qingru Hu:** data curation (equal), investigation (equal), writing – original draft (equal). **Rongping Bu:** writing – review and editing (equal). **Jiangbo Yang:** investigation (supporting). **Liu Lin:** conceptualization (equal), funding acquisition (lead), methodology (equal), project administration (equal), resources (lead), supervision (lead), writing – review and editing (equal). **Hai‐Tao Shi:** conceptualization (lead), funding acquisition (equal), methodology (lead), project administration (lead), resources (equal), supervision (equal).

## Conflicts of Interest

The authors declare no conflicts of interest.

## Supporting information


**Table S1.** Bayesian and Cliff’s Delta Results for Home Range Metrics and Male–Male Overlap.

## Data Availability

Due to ethical concerns regarding the conservation of an endangered species, the GPS data underlying this study are not publicly available. They can be shared by the corresponding author upon reasonable request and pending approval from the relevant conservation authorities.
