# Supplementary material for: Home Range of the Endangered Beale's Eyed Turtle ( Sacalia bealei ) and the Implications for Conservation
Source: Ecol Evol. 2025 Jun 11;15(6):e71520. doi: 10.1002/ece3.71520 (PMC12152767; doi:10.1002/ece3.71520)
Supplement: Supplementary file 1 — Table S1. Bayesian and Cliff’s Delta Results for Home Range Metrics and Male–Male Overlap. [file ECE3-15-e71520-s001.docx]

| Range Metrics | BF_10_ | | | Cliff's delta | |
| --- | --- | --- | --- | --- | --- |
|  | rscale=0.5 | rscale=0.707 | rscale=1.0 | δ | 95% CI |
| LHR (Year-round, m) | 0.76 | 0.68 | 0.58 | 0.44 | [-0.42, 0.89] |
| HR (Year-round, ha) | 0.89 | 0.82 | 0.74 | 0.44 | [-0.42, 0.89] |
| CHR (Year-round, ha) | 0.88 | 0.81 | 0.73 | 0.44 | [-0.42, 0.89] |
| LHR (Non-breeding. m) | 1.07 | 1.05 | 1.01 | 0.44 | [-0.73, 0.96] |
| HR (Non-breeding, ha) | 0.66 | 0.57 | 0.47 | -0.11 | [-0.79, 0.69] |
| CHR (Non-breeding, ha) | 0.67 | 0.58 | 0.49 | -0.11 | [-0.88, 0.82] |
| LHR (Breeding, m) | 1.11 | 1.10 | 1.07 | 0.78 | [-0.19, 0.98] |
| *HR (Breeding, ha) | 2.48 | 2.87 | 3.27 | 1 | [0.14, 1] |
| *CHR (Breeding, ha) | 2.06 | 2.32 | 2.56 | 1 | [0.14, 1] |
| Male-Male Overlap Index (Breeding vs. Non-breeding) | 0.8 | 0.73 | 0.65 | 0.33 | [-0.51, 0.85] |

Table S1. Bayesian and Cliff’s Delta Results for Home Range Metrics and Male-Male Overlap

*Significant results (BF_10_ ≥ 2 and CI excludes zero).
